# Bibliometric insights in fournier's gangrene: Research landscapes, turning points, and global trends

**DOI:** 10.3389/fsurg.2023.1057486

**Published:** 2023-02-16

**Authors:** Jia-Yuan Zhang, Chang-Fang Xiao, Chen Wang, Yi-Bo Yao

**Affiliations:** Department of Anorectal Surgery, Longhua Hospital, Shanghai University of Traditional Chinese Medicine, Shanghai, China

**Keywords:** fournier's gangrene, bibliometric analysis, visualization, global trends, research development

## Abstract

**Study Design:**

Bibliometric and visualization analysis.

**Objective:**

To analyze the research landscapes and hotspots of Fournier's gangrene, and reveal the dynamic changes and development trend of research hotspots for the purpose of providing ideas and a basis for clinical and basic research in this field.

**Methods:**

Research datasets were acquired from the Web of Science. The publication years were limited from January 1, 1900 to August 5, 2022. The bibliometric tools CiteSpace (v5.8) and VOSviewer (v1.6) were used to analyze the data and generate visualization knowledge maps. Trends in annual publications, distribution, H-index status, coauthorships status and research hotspots were analyzed.

**Results:**

According to the search strategy, we identified and enrolled 688 publications regarding to Fournier's gangrene. The number of published papers showed an overall upwards trend. The USA was the largest contributor, ranking first in total publications, citations and the H-index. The top 10 most productive institutions were all from the USA. De Simone B and Sartelli M were the most productive authors. There was close cooperation among countries, but the cooperation among institutions and authors had little contact and poor interactivity. The research hotspots included the pathogenesis factors and treatment. All the identified keywords were divided into 14 clusters, and the label of the latest cluster was “empagliflozin”. Prognosis and risk factors, emerging treatment methods, and pathogenesis were at the forefront of the Fournier's gangrene field and were predicted to be the next hot topics.

**Conclusion:**

The research of Fournier's gangrene has made some achievements, but the overall research level is still in the primary stage. The academic cooperation between different institutions and authors needs to be strengthened. At the early stage, the mainstream of research was the infected tissue and site, pathogenesis, and diagnosis of disease, while research on newly discovered sodium-glucose cotransporter 2 inhibitor, adjuvant therapy and prognostic factors may be the main directions in the future.

## Introduction

Fournier's gangrene (FG) is a rare, rapidly progressing and fatal infectious disease, with an incidence rate of approximately 1.6–3.3 cases per 100,000 males (1, 2). Most of the patients are men aged 60–70 (2, 3). The early clinical symptoms of FG include oedema, erythema and local tissue induration accompanied by fever. With aggravation of the infection, subcutaneous gas, pneumoscrotum, dyschromia, tissue necrosis, severe pain and foul smell gradually appear in the lesion area (4, 5). FG lesions are localized to the perianal and perineal triangle area at the beginning and can extend to the abdomen, thigh root, sacrococcygeal and lumbar back with inflammation (4, 6).

The typical symptoms of FG are present in only 10% to 40% of patients (7). It has characteristics of concealed onset, difficult diagnosis, rapid progression and powerful destruction. Delayed treatment options lead to serious complications and sequelae and even death. The mortality rate reported in the literature is approximately 20%–50%. However, the mortality rate increased to 70%–88% when patients were complicated with sepsis (1, 3, 8). Due to the rapid growth of modern intensive care treatment and modern medical technology, the mortality rate has decreased in recent years. Timely and effective treatment can reduce the mortality rate to less than 20% (2). Factors predisposing to the development of FG are oldness, diabetes, human immunodeficiency syndrome (HIV), kidney failure, cardiovascular disease, obesity, alcoholism and cancer (9–12). Patients with multiple risk factors are more likely to develop FG and have worse prognosis (11). Currently, the key to FG treatment is early and thorough surgical debridement, active use of broad-spectrum antibiotics and nutritional support (13).

In recent years, research on FG has been growing in scope, focusing on hyperbaric oxygen therapy, reconstructive surgery and mortality prediction models (6, 12, 14–17). However, they cannot directly reflect FG's research trajectory. Bibliometrics are scientific quantitative analysis methods that use mathematics, statistics and philology professional knowledge to analyze the current research achievement distribution (18, 19). This has played a great role in displaying the information panorama, key evolutionary pathways and future research hotspots of the field through quantitative analysis. This study aims to provide a research framework, relevant research status, and FG hot trends. Furthermore, the results also can provide some useful references for follow-up research.

## Materials and method

The Web of Science Core Collection database (WOS) is the most authoritative citation database in the world today. It has a powerful index function and collects influential core academic journals in various fields, including medicine. The relevant literature in the database reflects the latest development trends in this discipline or field in the world. In this study, relevant documents were retrieved from WOS. The search strategy was TS = “Fournier gangrene” OR “Fournier's disease” OR “Fourniers gangrene” OR “Fournier disease” OR “Perianal necrotizing fasciitis” OR “Perineal necrotizing fasciitis”. This search strategy retrieved records within English literature from January 1, 1900 to August 5, 2022. We obtain and store information such as article titles, abstracts, authors, keywords, research institutions, and reference data in a plain text format. All documents and information are retrieved and downloaded on the same day, avoiding unnecessary errors caused by daily database updates.

We retrieved a total of 668 data points, and the publication time span was from 1946 to 2022. Two researchers independently analyzed the data to ensure the accuracy and repeatability of the study. The original database was established with Microsoft Excel software, and the top cited articles, most productive countries/regions, authors, journals, and institutions were identified from the data.

In this study, the bibliometric tools CiteSpace (v5.8) and VOSviewer (v1.6) software were used to conduct scientific quantitative analysis on the number of articles, countries/regions, institutions, authors, journals, keywords and other key characteristics related to FG, and the original data were transformed into visualization. CiteSpace was used to reflect the number of annual publications and cocited authors/references. Through cocitation analysis, the development skeleton of FG was determined. CiteSpace has also been used to identify burst keywords that may reflect the annual research trends over the years (20). Coauthorship analysis was completed by VOSviewer, which was adopted to examine the cooperation relationship and closeness between authors, countries/regions and research institutions (21). In addition, VOSviewer was used for keyword co-occurrence analysis to identify research hotspots and predict future research trends.

## Results

### Trends in annual publication

**Figure 1:**
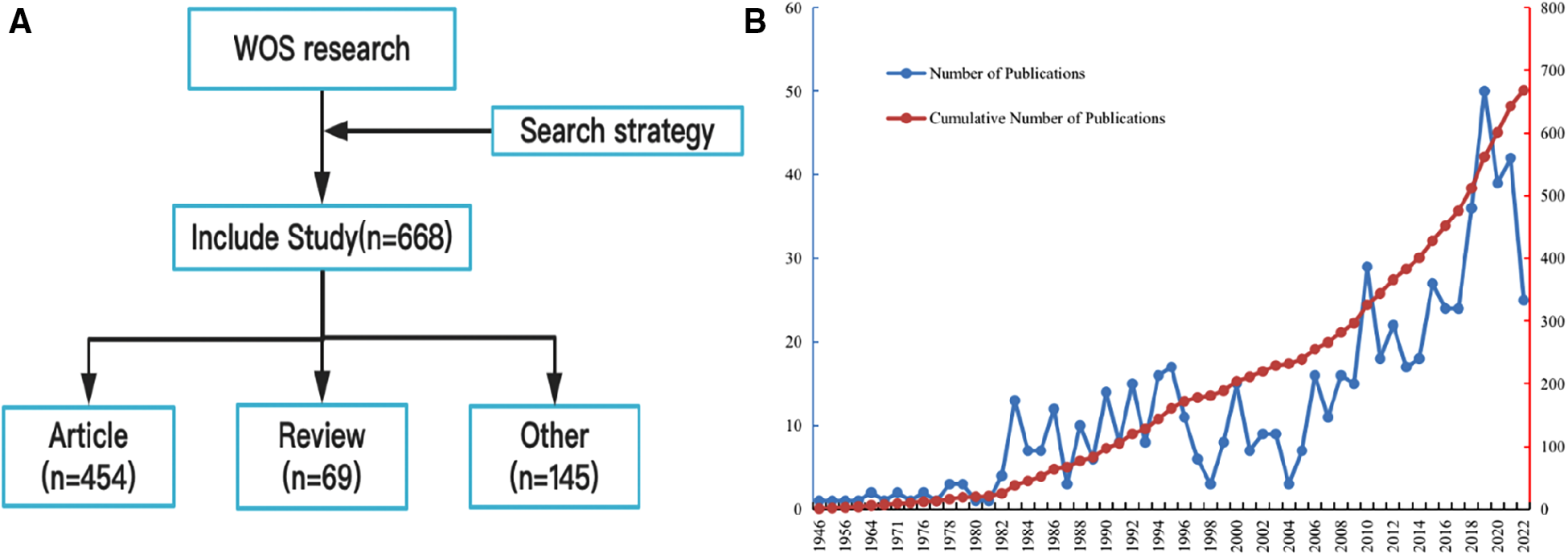
(**A**) Flow chart; (**B**) The annual trends of publications.

In total, we identified and enrolled 688 publications regarding FG, including 454 original articles, 69 reviews and 145 other types of publications (Figure 1A). The annual publications were important indicators to measure the development of this field. The annual publications from 1946 to 1981 were sporadically distributed, with number of 1–3 (Figure 1B). The number of publications issued from 1983 to 1997 fluctuated significantly, and the highest number (*n* = 17) issued at this stage appeared in 1995. From 1998 to 2022, publications related to FG showed fluctuating growth. Although there was a slight decline in individual years, the overall trend was upwards. Among them, the publications (*n* = 50) in 2019 exceeded a new high, and there was a slight decline in the next two years. This showed that FG received increasing attention from clinical and scientific researchers and entered a rapid development stage from the initial stage of research.

### Contributions of countries/regions

**Figure 2:**
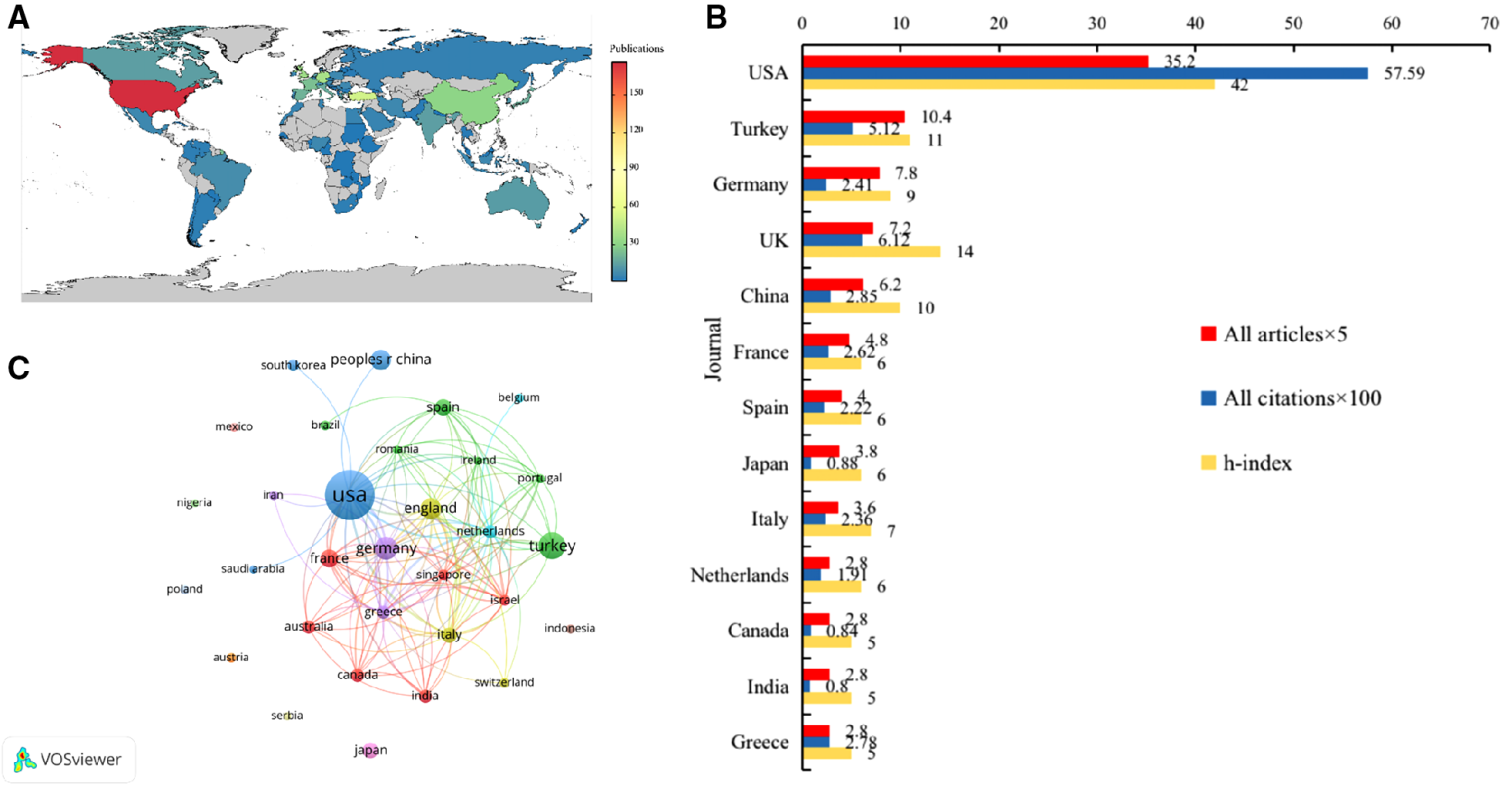
(**A**) The country-specific publication citation map; (**B**)Top 10 countries contributed to research publications in the field of FG; (**C**) The cooperation between countries/regions.

A country-specific publication citation map is shown in Figure 2A. The top 10 countries with the highest productivity are listed (Figure 2B and Table 1). The results showed that the USA published the largest number of FG-related studies, ranking first, with a total of 176 publications and accounting for 26.35%, followed by Turkey (*n* = 52) and Germany (*n* = 36). From the perspective of citation numbers, the USA still ranked first (*n* = 5759), far ahead of Britain and Turkey, which ranked second and third respectively. The H-index is a mixed quantitative index that can be used to evaluate the quantity and level of academic output (22). The USA had the highest H-index, followed by Britain and Turkey. The cooperation between countries/regions is shown in Figure 2C. The USA was the country with the most frequent participation in international cooperation, and China had the most cooperation with the USA.

**Table 1:** Top 10 countries contributed to research publications in the field of FG.

No.	Country	All articles
1	USA	176
2	Turkey	52
3	Germany	39
4	UK	36
5	China	31
6	France	24
7	Spain	20
8	Japan	19
9	Italy	18
10	Netherlands	14

### Active institutions

**Figure 3:**
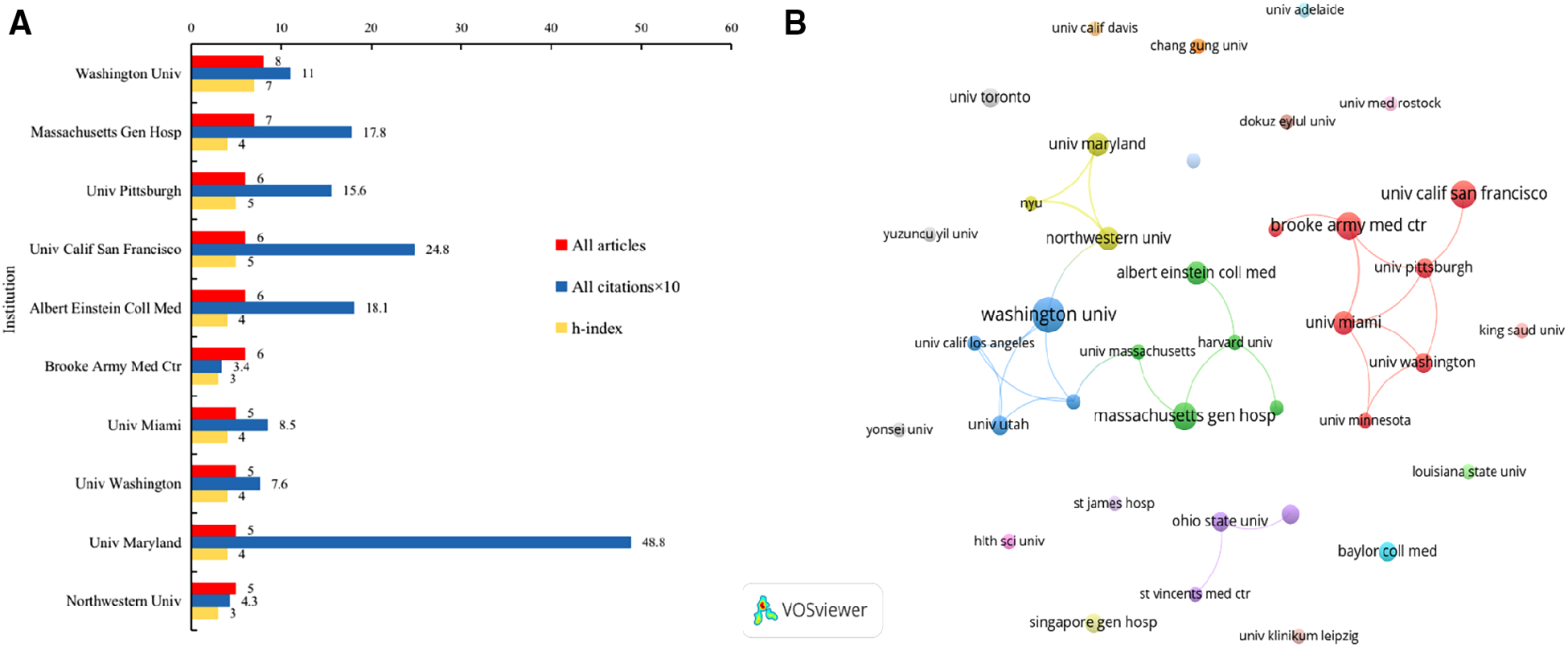
(**A**) Top 10 institutions contributed to research publications in the field of FG; (**B**) The cooperation between institutions.

The top 10 institutions with the most publications are shown in Table 2 and Figure 3A. Washington University contributed the maximum number of publications (*n* = 13), while Massachusetts General Hospital (*n* = 7) ranked 2nd. The University of Pittsburgh, University of California, San Francisco, Albert Einstein College of Medicine and Brooke Army Medical Center ranked 3rd, each with 6 published articles. The papers published by the University of Maryland received the highest citations, followed by the University of Port Harcourt and the Charles McC Mathias Jr. National Study Center for Trauma and Emergency Medical Systems. In terms of the H-index, Washington University ranked first (*n* = 11), followed by the University of California, San Francisco (*n* = 6), and the University of Pittsburgh (*n* = 6). The top 10 most productive institutions were all from the USA.

**Table 2:** Top 10 productive institutions in the field of FG.

No.	Institution	All articles
1	Washington Univ	13
2	Massachusetts Gen Hosp	7
3	Univ Pittsburgh	6
4	Univ Calif San Francisco	6
5	Albert Einstein Coll Med	6
6	Brooke Army Med Ctr	6
7	Univ Maryland	5
8	Univ Miami	5
9	Northwestern Univ	5
10	Univ Utah	4

The institutional cooperation network map is shown in Figure 3B. The links between nodes in the map represent the cooperation relationships. The distance between the nodes and the thickness of links indicated the cooperation levels between productive institutions. There was close cooperation among the top 10 institutions, and 4 major subcooperation networks were formed.

### Productive journals and cocitation analysis

**Figure 4:**
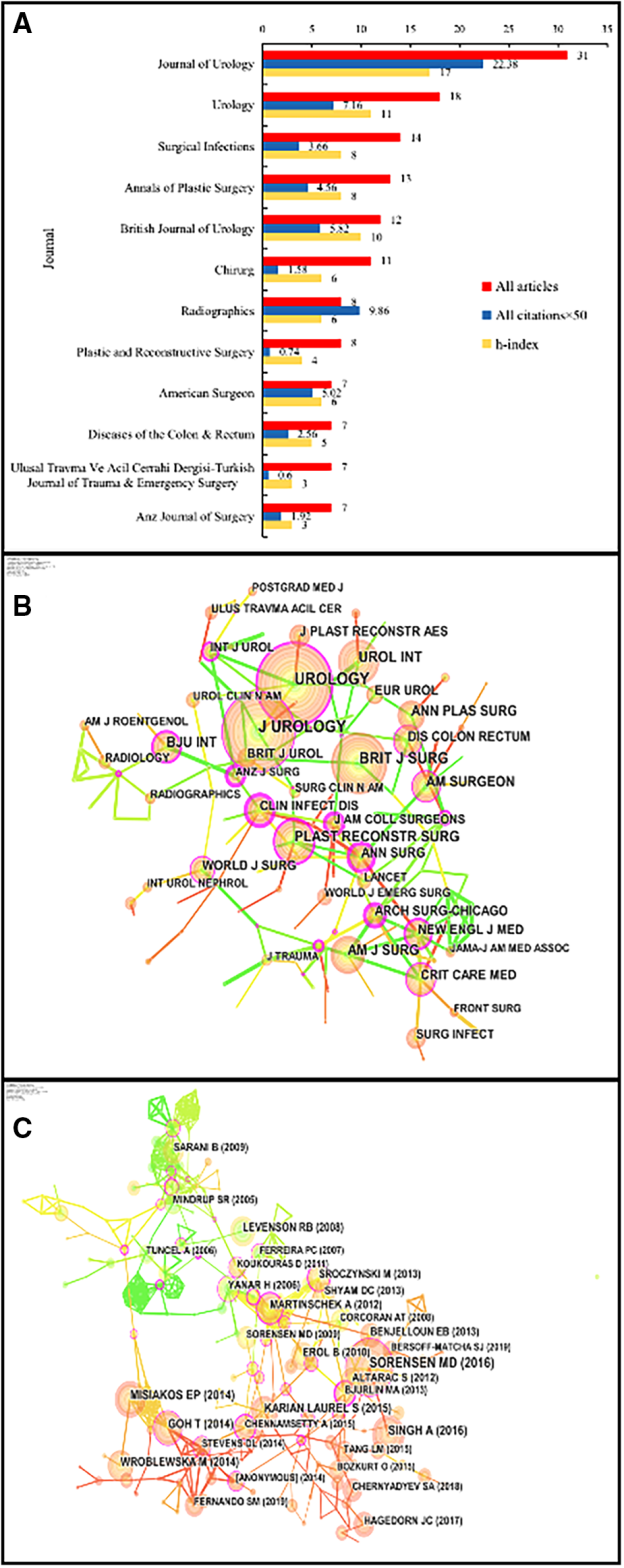
(**A**) Top 12 journals in the field of FG; (**B**) The co-cited map of the journals. (**C**) The keyword co-occurrence analysis.

We listed the 12 most productive journals in this field in Table 3 and Figure 4A. In addition to ranking first in the number of contributing papers (*n* = 31), the Journal of Urology was also the journal with the highest citation and H-index. Urology (*n* = 18) and Surgical Infections (*n* = 14) ranked 2nd and 3rd, respectively. We provided a cocitation map of the journals in Figure 4B. The cocitation frequency of the Journal of Urology (*n* = 157) and Urology (*n* = 156) was not much different, which indicated that the quality of the articles published by these two journals in the FG field was good.

**Table 3:** Top 12 journals in the field of FG.

No.	Journal	All articles
1	Journal of Urology	31
2	Urology	18
3	Surgical Infections	14
4	Annals of Plastic Surgery	13
5	British Journal of Urology	12
6	Chirurg	11
7	Radiographics	8
8	Plastic and Reconstructive Surgery	8
9	American Surgeon	7
10	Diseases of the Colon & Rectum	7
11	Ulusal Travma Ve Acil Cerrahi Dergisi-Turkish Journal of Trauma & Emergency Surgery	7
12	Anz Journal of Surgery	7

If two or more articles were cited by one or more later articles at the same time, it was said that these two or more articles had a cocited relationship (23). We found that there were 31 publications with a cocitation frequency of 10 or more. Among them, the top cited publication was published in the International Journal of Urology in 2016 by Sorensen MD, and it achieved 28 citations. The cocitation frequency of the other 9 papers was 14–20. We show the cocitation network in Figure 4C. There were 274 nodes and 660 links in the network, with a density value of 0.0176. It could be roughly divided into three subnetwork structures centred on the publications of Sorensen MD, Goh T and Martinschek A.

### Contribution of authors

To a certain extent, the number of publications reflects the author's academic ability and scientific research level. Since there were 21 authors whose number of publications was 3, we listed the top 23 authors with the most publications in Table 4. De Simone B and Sartelli M were the most productive authors (*n* = 4). Eke N was the author with the most citations (*n* = 429), followed by Myers Ram, Kufera JA, and Elliott DC (*n* = 411). For the H-index, De Simone B, Sartelli M, Menias CO and Mellnick VM shared first place. In addition, we made an author collaboration network map. Several authors' subnetwork structures were formed in the map. The connections of subnetwork structures were sparse, indicating that there was relatively little cooperation among different authors in this field, and the cooperation density was not strong. There was a lack of awareness of communication and cooperation among scholars in the field of FG in various countries. Academic exchanges and cooperation among scholars need to be strengthened.

**Table 4:** Top 23 productive authors in the field of FG.

No.	Author	All articles
1	De Simone B	4
2	Sartelli M	4
3	Menias CO	3
4	Mellnick VM	3
5	Coimbra R	3
6	Coccolini F	3
7	Tarasconi A	3
8	Ansaloni L	3
9	Sen V	3
10	Kluger Y	3
11	Long B	3
12	Leppaniemi A	3
13	Lehnhardt M	3
14	Kujath P	3
15	Litvin A	3
16	Catena F	3
17	Sahin MO	3
18	Di Saverio S	3
19	Daigeler A	3
20	McAninch JW	3
21	Karaca C	3
22	Kim DY	3
23	di Summa PG	3

### Research hotspots and frontiers in this field

**Figure 5:**
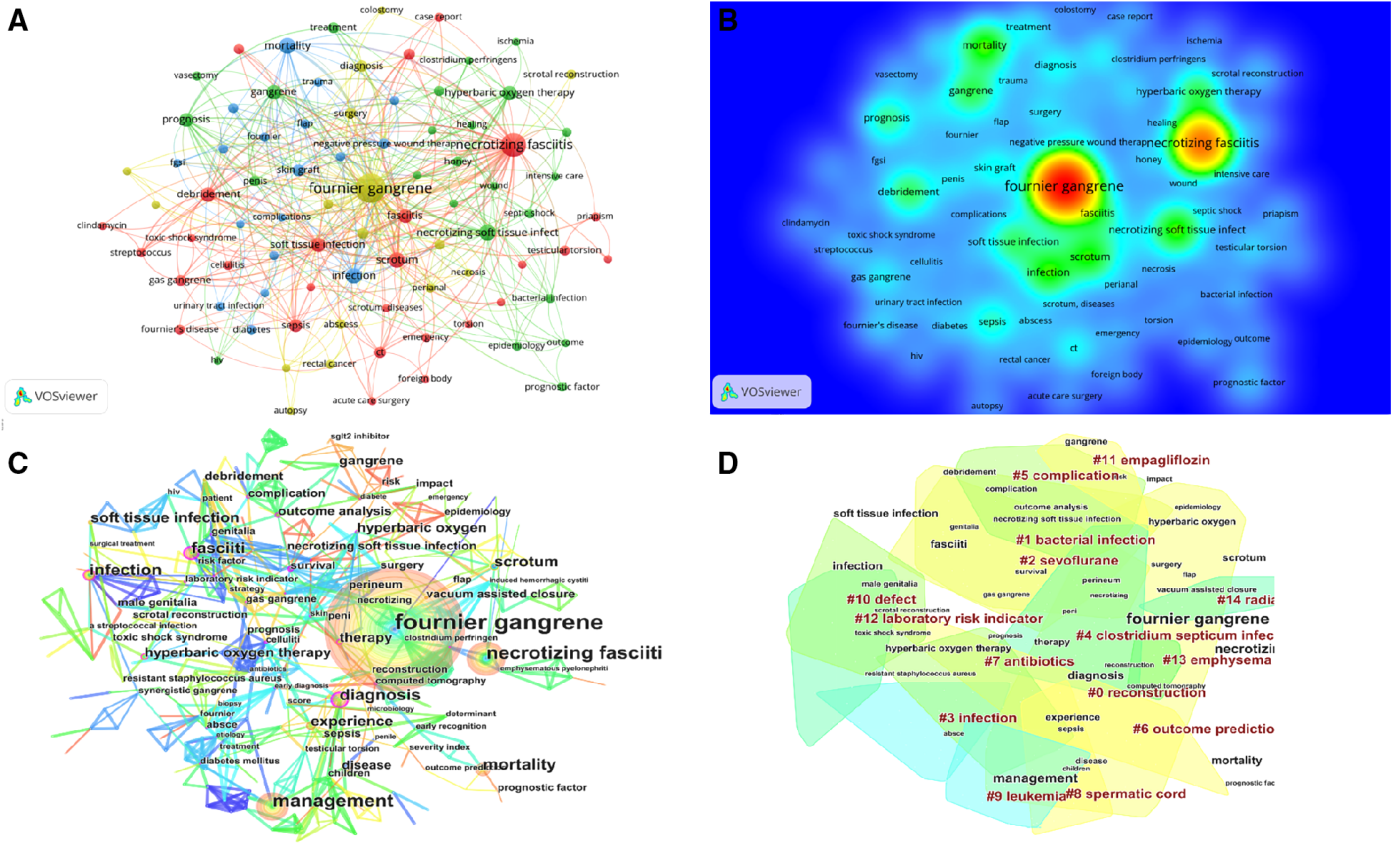
(**A**) The keyword network map; (**B**) The keyword hotspot map; (**C**) The keyword co-occurrence analysis; (**D**) The 14 keyword clusters.

The top 10 high-frequency keywords in the FG research field are shown in Table 5. Figure 5A shows the keyword network map, which visualizes the research hotspots in this field. In addition to “Fournier gangrene”, other closely related high-frequency keywords included “scrotum”, “necrotic soft tissue infection”, “sepsis”, “debridement”, “mortality”, “hyperbaric oxygen therapy” and “prognosis”, which were consistent with the hotspot map (Figure 5B).

**Table 5:** The top 10 high-frequency keywords in the field of FG.

No.	Freq	Year	Keywords
1	381	1991	Fournier gangrene
2	135	1991	Necrotizing fasciiti
3	113	1993	Management
4	67	1991	Infection
5	63	1999	Diagnosis
6	62	2000	Mortality
7	54	1992	Fasciiti
8	49	1992	Scrotum
9	44	1994	Soft tissue infection
10	39	2000	Experience

Through further calculation of high-frequency keywords, 5 keywords with centrality greater than 0.2 were obtained: “fasciiti”, “diagnosis”, “computed tomography”, “survival” and “celluliti”. The higher the centrality is, the greater the attention of researchers, the deeper the influence and the longer the duration of research in this field. These central keywords represented the research directions with high attention in the field of FG research. The results of keyword co-occurrence analysis showed that the research in the FG field mainly focused on infection, diagnosis and treatment (Figure 5C). Among them, the research on hyperbaric oxygen was the most extensive and in-depth.

All the identified keywords were divided into 14 clusters, and the cluster names were refined according to the keywords contained in each cluster (Figure 5D). These clusters covered the main hot research directions of the FG field. The higher the size value is, the higher the popularity of the clustering research. The higher the silhouette value is, the higher the consistency among the members of the cluster. Further analyzing the knowledge structure of these 14 clusters (Table 6), we found that Cluster #0 had the highest size value (42) and was labelled “reconstruction”. We found that Cluster #3 had the largest silhouette value (0.941) and was labelled “infection”. We found that the latest cluster was #11, whose average year was 2016, and the label was “empagliflozin”.

**Table 6:** The 14 clusters of keywords in the field of FG.

ID	Size	Silhouette	Year	Label (LLR)
0	36	0.838	2003	Reconstruction; epididymiti; torsion
1	31	0.856	2004	Bacterial infection; epidemiology; critical care
2	30	0.862	2004	Sevoflurane; clinical feature; invasive
3	28	0.941	1998	Infection; etiology; biopsy
4	24	0.913	2000	Clostridium septicum infection; acute promyelocytic leukemia
5	22	0.811	2006	Complication; outcome analysis; penile lesion
6	21	0.851	2009	Outcome prediction; prognostic factor; mortality
7	21	0.908	2001	Antibiotics; colorectal disease; acid
8	20	0.938	2008	Spermatic cord; children; color doppler sonography
9	19	0.92	1999	Leukemia; group G streptococcus; perirectal infection
10	17	0.94	2000	Defect; male genitalia; acute renal failure;
11	17	0.876	2016	Empagliflozin; SGLT-2 inhibito; risk
12	17	0.94	2007	Laboratory risk indicator; resistant staphylococcus aureus
13	13	0.94	2001	Emphysema; prognostic value; gastriti
14	13	0.915	2002	Radiation cystiti; induced hemorrhagic cystiti; osteoradionecrosis

**Figure 6:**
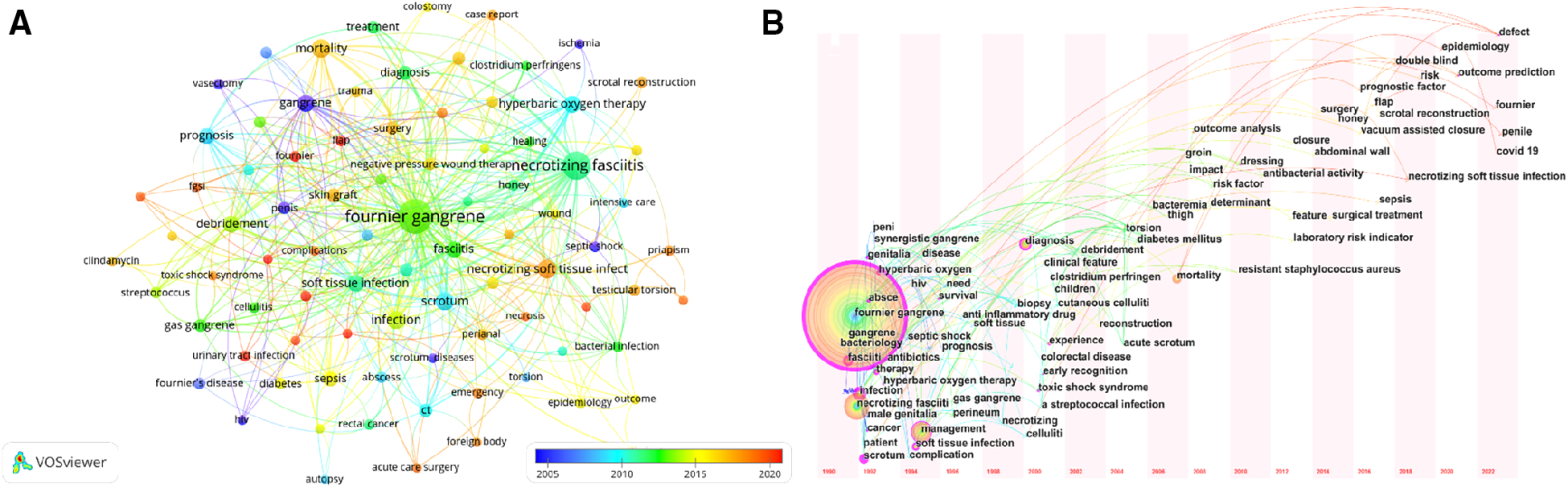
(**A**) The keyword-cluters timeline map; (**B**) The keyword timeline view.

The keyword timeline view of the publications is shown in Figures 6A,B. Over time, increasing attention has been given to the overall prognosis of patients, including the development of new adjuvant therapies and prognostic factors. The emergence of keywords was the change rate of the cited frequency of keywords, which represented that the keywords were widely concerned in a short period of time and often became the research hotspot in the future (23). It was used to study the dynamic development and potential problems of a certain field and was suitable for testing the new trends and sudden changes in the development of disciplines. Table 7 lists the top 20 keyword bursts and shows the burst intensity and the start or end year. According to the information in the table, the early research focus was on the site of the disease (male genitalia), which became the focus of research from 1990 to 2002. Subsequently, antibiotics (penicillin), hyperbaric oxygen and other treatment methods began to receive attention. At the same time, scholars began to study the impact of HIV and other immune deficiency states on the occurrence and development of the diseases. In the middle stage, the research focus shifted to early recognition and debridement. In recent years, prognosis and risk factors, emerging treatment methods (vacuum assisted closure), and pathogenesis (soft tissue infection, sepsis and sodium-glucose cotransporter 2 inhibitor) have been at the forefront of the FG field and may be a future research direction.

**Table 7:** The top 20 keywords burst.

Keywords	Strength	Begin	End	1990–2022
Genitalia	6.9009	1990	2002	——————————————————————————————————
*P*eni	4.8057	1992	2005	——————————————————————————————————
Hyperbaric oxygen therapy	7.7911	1992	2002	——————————————————————————————————
Synergistic gangrene	3.8986	1992	2000	——————————————————————————————————
Hiv	2.9561	1994	2002	——————————————————————————————————
Early recognition	2.4822	1997	2009	———————————————————————————————————
Children	2.6976	2001	2004	——————————————————————————————————
Experience	4.2648	2007	2018	——————————————————————————————————
Therapy	2.9431	2009	2012	——————————————————————————————————
Debridement	2.4898	2010	2012	—————————————————————————————————
Resistant staphylococcus aureus	3.9857	2010	2017	——————————————————————————————————
Surgical treatment	2.7895	2012	2015	——————————————————————————————————
Prognostic factor	2.7519	2012	2022	——————————————————————————————————
Complication	2.9405	2014	2016	——————————————————————————————————
Mortality	3.4519	2014	2022	——————————————————————————————————
Soft tissue infection	3.221	2015	2018	——————————————————————————————————
Vacuum assisted closure	4.509	2015	2022	——————————————————————————————————
Sepsis	4.1236	2016	2022	——————————————————————————————————
sglt2 inhibitor	3.0448	2019	2022	——————————————————————————————————
Outcome analysis	4.5569	2020	2022	——————————————————————————————————

## Discussion

### Trends of publications related to FG

Based on the relevant literature in the FG field collected by WOS, this study analyzed the literature publication trends, main research content, research hotspots and future research directions of FG by bibliometrics and visual analysis.

Although the number of FG-related papers published annually fluctuated, it was generally on the rise. Especially in 2019, there was the maximum with 50 publications. This shows that the researches on emergency and severe cases in the global medical field has deepened and expanded with the rapid development of modern medical technology and intensive care treatment. However, at the same time, compared with other disciplines, research on FG is still relatively limited and requires continuous long-term development and exploration.

This study found that the number of publications, citations and the H-index in the USA were far higher than those in other countries. In addition, the top 10 institutions were all from the USA. The above results showed that the research on FG is mainly concentrated in the United States, and other countries need to strengthen their FG research and cooperation with the USA. We believe the most likely reason is related to its huge technical, economic and academic advantages and the large amount of research funds invested in medical research.

In terms of journals, the Journal of Urology was undoubtedly the leader, ranking first in terms of articles, citations and the H-index. It is worth noting that although Urology ranks lower than the Journal of Urology in the above aspects, in the cocitation map, Urology had a comparable cocitation frequency to that of the Journal of Urology (156 vs. 157), and the centrality was higher than that of the Journal of Urology (0.23 vs. 0.2). Researchers interested in FG need to pay more attention to the contents of these two journals.

From the perspective of the network map of cooperation relations, there was close cooperation among countries, but cooperation among institutions and authors had poor interactivity. This suggested that further strengthening the cooperation among institutions and authors, expanding the scope of cooperation, and forming an academic community are important aspects to promote the deepening and accelerating development of FG research in the future.

### Studies focused on FG

Through keyword co-occurrence, cluster analysis, timeline view analysis and emergence analysis, it was found that the main research directions of FG include lesions, pathogenic factors and management.

In 1883, French dermatologist Jean Alfred Fournier (1832–1914) reported and described five young male patients with “explosive scrotal gangrene” (24). Since then, the term “Fournier gangrene” (FG) has gradually been accepted. During this period, terminology such as “periurethral cellulitis, synergistic gangrene, and gangrenous pyoderma” had also been included in the concept of this disease. In recent years, research in this field has gradually increased. Meanwhile, scholars have gradually realized that this disease is closely related to the urogenital tract and ano-rectum source (25). At present, it is believed that only approximately one-quarter of cases are idiopathic diseases with unknown causes, and more than 75% have definite aetiologies (26, 27). As for the infection site, 30%–50% originate from the rectum and anus, 20%–40% originate from the urogenital system, and 20% originate from skin infection (28). Why is there only “genitalia” but no “perianal” or “perineum” in the top 20 burst words? On the one hand, the early manifestation of the disease is nonspecific, and ano-rectal source FG is often mistaken for a perianal abscess. In recent years, the two have been gradually differentiated, and there is a deeper understanding of ano-rectal source FG with the progress of medical research. At the same time, the infection in FG tends to spread along fascial planes (9). The deep layer of superficial perineal fascia, Colles’ fascia, is continuous with the Dartos fascia of the scrotum and the Scarpa fascia of the abdominal wall (29). Therefore, infection can spread *via* these routes. As ano-rectal source FG can break through the Colles' fascia forwards or upwards through the surrounding infection, it is usually accompanied by the appearance of genitalia. The most frequent sites of origin of infection were the scrotum and perineum (26). On the other hand, the urogenital source of infection instead of the ano-rectal source was significantly associated with a higher mortality rate (25). This may be the reason why researcheres pay more attention to the “genital” part.

The second research direction of FG was pathogenic factors. Aerobic bacteria and anaerobic bacteria exist at the same time in patients with FG. When the host has immunodeficiency, a polymicrobial flora are usually involved with a synergic mechanism of aggressiveness (28). It was reported 54% of case were caused by Polymicrobial infection (30). The pathogenic bacteria are mainly gram-negative bacteria, most commonly Escherichia coli, Streptococcus, Klebsiella pneumoniae, Staphylococcus, Pseudomonas aeruginosa and Vibrio vulnificus (8, 30, 31).

The predisposing factors to FG include diabetes, human immunodeficiency virus (HIV), renal failure, liver cirrhosis, malignant tumors, smoking and alcohol consumption (7). These are conditions that lead to compromised host immunity, all of which create an environment that is favourable for establishing infection. In patients who present with FG, the rate of diabetes mellitus is estimated to be 32%–66% (32–35). Hyperglycemia will affect the adhesion, chemotaxis and fungicidal effectiveness of white blood cells, leading to impaired immune response and increasing the risk of sepsis in patients (8). HIV was reported to be the most common potential pathogenic factor of FG in HIV era endemic areas such as Africa (36, 37). As confirmed by other reports, 4% of FG patients were found to be complicated with HIV infection at the time of admission (35, 37). In our research, HIV emerged from 1994 to 2002 (Table 7). Alcoholism is present in 20%–50% of patients with FG (12, 33). Many reports have demonstrated an association between deteriorated renal function and the incidence of FG (38).

More recently, researchers have noticed that sodium-glucose cotransporter 2 (SGLT-2) inhibitor may lead to the occurrence of FG. This conclusion can be confirmed with keyword co-occurrence analysis, cluster analysis and emergence analysis. SGLT-2 inhibitor reduces blood glucose by inhibiting glucose reabsorption in the proximal tubules, leading to increased urine glucose and elevating the risk of genitourinary system infection (39, 40). Bersoff analyzed the U.S. Food and Drug Administration (FDA) Adverse Event Reporting System and published case reports identifying 55 cases of FG in patients receiving SGLT-2 inhibitors between 1 March 2013 and 31 January 2019 (41). Obtaining SGLT-2 inhibitors may be a possible predisposing factor for FG. Some people have doubts about this view, because diabetes is a risk factor for the onset of FG (8). By comparing and analysing patients who used SGLT-2 inhibitor and other types of hypoglycaemic drugs, it was identified that SGLT-2 inhibitor did not improve the incidence rate of FG (42, 43). We note that since the FDA issued the warning that “FG may be related to new oral anti-diabetic drugs called SGLT-2 inhibitors” in 2018, the relationship between the two has become a research hotspot (44). However, bibliometric results indicate that relevant studies are typically conducted primarily in the United States and that they are case reports or retrospective studies with small sample sizes. Therefore, it is urgent that other countries join this study and carry out an investigation. Multicentre or multiregion studies with large sample sizes may be more able to verify whether there is a causal relationship between FG and SGLT-2 inhibitors.

The third research direction of FG was management. The key to treating FG lies in early diagnosis and timely treatment. The main treatment principles of this disease are early, active and thorough surgical debridement and combined use of broad-spectrum and sufficient antibiotics (13). Debridement should be performed as soon as possible for patients with clinical suspicion. If the severity of the disease is not recognized because of the inconspicuous early clinical manifestation, the initial debridement will be delayed, and definitive treatment will be held up. Delayed treatment leads to a significant increase in the mortality rate of patients. Kalaivani observed that debridement performed 24 h after admission was an independent risk factor for a high case fatality rate (45). Wong showed that debridement 24 h after the completion of the evaluation could lead to a 9-fold increase in mortality (46). Early debridement is the key to successful treatment. In Table 7, “surgical treatment” ranked 16th, “debridement” ranked 19th, and “early recognition” ranked 20th. Aggressive debridement may cause significant tissue loss, which prolongs the healing process, causing longer hospital stays and higher hospitalization expenses. Therefore, after active debridement and drainage, doctors often use adjuvant treatment to help patients reduce pain and allow the healing process to accelerate.

New adjuvant treatments currently include hyperbaric oxygen therapy and vacuum sealing drainage. In Table 7, “hyperbaric oxygen therapy” (HBOT) was the keyword with the highest emergence strength and became a research hotspot from 1992 to 2002. A retrospective review of a large nationwide database (*n* = 45913) reported that patients receiving HBOT had a significant reduction in mortality (4.5% vs. 9.4%, *P *= 0.001) (47). Assen conducted a retrospective analysis over 10 years, including 192 patients, and grouped patients according to the indications of HBOT for the first time. The survival rates of the two groups were similar, but the baseline conditions of the group receiving HBOT treatment were significantly worse, and the scope of lesions was larger (48). Arterial thrombosis causes hypoxia, which leads to tissue ischaemia and necrosis and creates a favourable environment for the propagation of anaerobic bacteria. Using HBOT after debridement can increase the oxygen content of the tissue, inhibit anaerobic bacteria, relieve inflammatory responses (47, 49). During later stages, HBOT can improve angiogenesis, fibroblast activity, and collagen synthesis, thus promoting wound repair and healing (12, 47).

“Vacuum assisted closure” (VAC) has been a burst keyword since 2015 and is still a research hotspot. Valerio conducted a multicentre retrospective analysis and found that disseminated FG patients treated with VAC therapy had a higher 10-week wound closure cumulative rate of approximately 75% (50). Daniela found that VAC caused fewer dressing changes, less pain, and less need for analgesics but a higher length of hospital stay through a systematic review (51). It is believed that VAC is an effective way to promote the healing of surgical wounds and complicated wounds that fail primary healing. When open wounds are exposed to negative pressure, they can reduce tissue oedema and increase blood flow, thus promoting healing (35).

The main treatment of FG is extensive debridement and resection, which often leads to large tissue loss and poor appearance. Once the necrotic tissue of the patient has been eliminated and the potential infection has been systematically controlled, genital and perineum reconstruction surgery can be considered (6). The primary goal of reconstruction is simple and efficient coverage (12). In addition, restoring functionality, shortening the length of hospital stay and improving the psychological condition of the patients are essential (6, 27, 52). At present, there are several reconstructive surgical techniques, including skin grafts, transposition of the testicles and spermatic cords to a subcutaneous pocket in the upper thigh, fasciocutaneous flaps and several other types of pediculated myocutaneous flaps (53, 54). The multiplicity of techniques demonstrates that there is potentially no single favored reconstructive technique (55), and most scholars believe that flaps give superior cosmetic aspects (27, 53, 56, 57). In Figure 5D and Table 6, “reconstruction” was one of 14 keywords clusters and had the highest size value, which proved that reconstruction had been a hot topic in FG research and deserved our continued attention.

As medical standards improved, an increasing number of scholars begun to pay attention to the complications, prognosis and clinical outcomes of FG patients. Table 7 and Figure 5D provide evidence for the above viewpoint. Accurate evaluations of the patient's condition at admission can improve the alertness of medical staff in the diagnosis and treatment of diseases, promote early identification of serious patients and early surgical treatment, relieve the occurrence of complications, improve the prognosis of patients, and reduce mortality. Because the evaluation scale based on laboratory indicators is simple and convenient, it is often used by doctors as a disease evaluation tool. The FGSI is the most commonly used prognostic assessment scale and research hotspot to date (Figure 5D). In addition, scholars have also developed scales such as UFGSI, ACCI and LRINEC, but the external validation has different evaluations of the sensitivity and specificity of the above scales (58–60).

### Strengths and limitations

We conducted a comprehensive and objective search of the relevant FG literature through WOS to obtain reliable results and provided some insights into the research characteristics and article citations in the FG field. However, this study still has many limitations. First, this study included only articles in WOS. However, Embase, Cochrane Library and other databases were not searched, and unpublished articles were not included. Second, the results of bibliometrics may be different from the actual research. For example, some recently published high-quality literature may not be considered because of low citation frequency. Therefore, it is still necessary to continue to pay attention to the latest published literature. Third, the bibliometric data will change with time, and the delay of indexation leads to partial changes in the results. Fourth, we did not read the contents of the article one by one. Whether it was cited for a positive contribution, negative impact or even poor quality, we included all of them.

## Conclusions

Due to its insidious onset, strong invasiveness and high mortality, FG has brought great challenges to health care staff engaged in this field. We used bibliometrics to visually analyze the literature data of FG to evaluate past research activities, track research progress, and predict discipline dynamics. The annual number of publications issued in this field fluctuated slightly, but the overall trend was upwards. There was a lack of cooperation between the authors and various research institutions. It is necessary to strengthen cooperation and exchange to promote more comprehensive and systematic research. From the perspective of research hotspots and development trends, in the early stage, lesions, pathogenesis and early diagnosis attracted the attention of scholars. With the passage of time, significant progress has been made in the study of newly discovered risk factors, emerging adjuvant treatments and prognostic factors, which are at the forefront of this field and point out the directions for future research.

## Data Availability

The original contributions presented in the study are included in the article/Supplementary Material, further inquiries can be directed to the corresponding author/s.
