# Supplementary material for: Bibliometric insights in fournier's gangrene: Research landscapes, turning points, and global trends
Source: Front Surg. 2023 Feb 16;10:1057486. doi: 10.3389/fsurg.2023.1057486 (PMC9978006; doi:10.3389/fsurg.2023.1057486)
Supplement: Supplementary file 1 [file Datasheet1.zip › Supplementary_Material/Supplementary_Material.docx]

Supplementary Material

# Supplementary Data

## Publications Regarding FG

### 688 Publications

### Annual Publications

## Contributions of Countries/Regions

### Contributions of Countries/Regions

### Countries/regions Cooperation

## Contributions of Institutions

### Contributions of Institutions

### Institutional Cooperation

## Contributions of Journals

### Contributions of Journals

### Cocitation if the Journals

## Contribution of Authors

### Contribution of Authors

### Author Collaboration

### Cocitation of the Authors

## Cocitation Analysis

## Keywords Analysis

### Keywords Frequency

### Keywords Clusters
